# Development and Validation of the Primary School Students’ Perceived Teacher Trust Behaviors Scale

**DOI:** 10.3390/bs16010074

**Published:** 2026-01-06

**Authors:** Yao Wang, Jie Chen, Guangming Li, Xiaofeng Zheng, Xuelan Liu

**Affiliations:** 1 School of Education, South China Normal University, Guangzhou 510631, China; 2023010055@m.scnu.edu.cn; 2 Institute of Education Sciences, Qiannan Normal University for Nationalities, Duyun 558000, China; 3 Center for Studies of Psychological Application, Guangdong Key Laboratory of Mental Health and Cognitive Science, School of Psychology, South China Normal University, Guangzhou 510631, China; 4 Key Laboratory of Brain, Cognition and Education Sciences, Ministry of Education, South China Normal University, Guangzhou 510631, China

**Keywords:** teacher trust, student perception, scale development, psychometric validation, primary education

## Abstract

This study aimed to develop and validate a self-report instrument—the Perceived Teacher Trust Behavior Scale (PTTBS)—to assess primary school students’ perceptions of trust-related behaviors exhibited by their teachers. Adopting a child-centered perspective within the school context, we first conducted in-depth interviews and applied a grounded theory approach to identify dimensions and generate initial items. A cluster sampling method was used to recruit 1400 students (Grades 3~5) from three schools in Guizhou Province, China, who completed the questionnaire. The collected data were analyzed via exploratory factor analysis and confirmatory factor analysis using SPSS 30.0 and Mplus8.10 software. The final version of the PTTBS consists of 13 items across four dimensions: Emotional Support, Competence Recognition, Academic Support, and Moral Recognition. The scale demonstrated excellent internal consistency (Cronbach’s α = 0.90) and split-half reliability (Spearman–Brown coefficient = 0.847). Significant correlations with an established Student-Teacher Relationship Scale were observed, along with good convergent validity (0.502~0.629) and construct validity. The PTTBS exhibits robust psychometric properties and serves as a valid tool for measuring Chinese primary school students’ perceptions of teacher trust behaviors, suitable for both research and practical applications.

## 1. Introduction

Trust is a pervasive and essential element in the development and relationships of human societies. Without a degree of trust and shared meaning, it would be impossible to establish sustained and stable social relations (Eisenstadt & Roniger, 1984). The composition and evolution of human societies are primarily constituted by biologically unrelated “strangers” (Gächter & Fehr, 1997). In today’s context, characterized by contingency, challenges, volatility, and globalization, trust has become an urgently central issue. To ensure the smooth functioning of such societies, interpersonal trust is crucial amid modern social transformations (Rotenberg, 2019). Psychologist Deutsch (1958) emphasized that interpersonal trust refers to an individual’s or group’s generalized and reliable expectation regarding the credibility of others’ words, promises, and oral or written statements. Its development is rooted in early childhood socialization environments, where children, through social learning processes in cooperative and supportive social settings, gradually form heightened “trust expectations” toward others or groups.

Trust plays a critical role in shaping a child’s personality (Zhao et al., 2024), social adjustment (M. Li et al., 2025), and intellectual development (Bernath & Feshbach, 1995). Within educational contexts, trust is regarded as a fundamental relational dynamic and a synthesizing force that underpins pedagogical interactions (Hektoen, 2025). Conceptualized as a form of social capital (Moon & Coleman, 1990), trust plays an instrumental role in motivating the trusted individual, thereby stimulating and unlocking their intrinsic initiative and agency (Sztompka, 1999/2005). From a sociological perspective, Luhmann (1968/2005) contends that trust is invariably embedded within social relations, which are themselves governed by specific rule systems. Within these interactive frameworks, trust emerges under the influence of both psychological and social factors and cannot be attributed solely to one individual. Anthony Giddens (1990/2000), from an ontological standpoint, defines trust as the confidence in the reliability of a person or system. This framework is particularly useful for understanding the ethical foundations of teacher-student trust relationships. Giddens distinguishes between interpersonal trust, based on the other’s virtue (e.g., honesty, kindness), and system trust, grounded in confidence in the correctness of principles (C. Fu & Zhao, 2019). Furthermore, rational choice theory posits that interpersonal trust is a prerequisite for functional interpersonal relationships (Becker, 1974; White, 2023).

Bronfenbrenner and Morris (2007), in their research on interactions within the microsystem of roles and relationships, introduced the concept of “significant others” to describe individuals with whom a child regularly interacts over a sustained period, primarily parents, teachers, and friends. Theories of significant others suggest that among myriad social environmental factors, an individual’s interaction with these key figures plays a pivotal role in fulfilling basic psychological needs (Xu et al., 2025). Empirical studies indicate that perceiving trust from significant others, such as parents and teachers, can significantly predict self-esteem levels in middle school students (G. Fu, 2025). This perception creates a positive feedback loop that activates relevant brain regions and enhances self-worth (Chen et al., 2020). Perceived teacher trust has a significant and positive impact on student learning (e.g., in mathematics and reading), and its influence even exceeds the negative effects of social economic status (SES), The effect of perceived teacher trust on student learning is greater during the elementary school stage than in other learning stages (Sun et al., 2023). Furthermore, perceived teacher trust is a significant predictor of student gratitude (Yao, 2024), moral decision-making (Huang, 2023), and moral identity (Wang et al., 2020; Lian, 2024). This perceived trust is a key variable influencing teacher-student relationships (Sutherland et al., 2024). Positive teacher-student relationships, in turn, are conducive to the development of students’ moral character and academic achievement, fostering positive personality traits and stronger social adaptation skills (Aldhafri & Alhadabi, 2019). Such relationships also promote social-emotional competencies in primary school students (Deng et al., 2022), prosocial behaviors like gratitude (Wu & Zhang, 2022), and can positively predict children’s moral sensitivity (Yang et al., 2025).

It should be further clarified that the “Student-Teacher Relationship Scale” developed by Pianta (2001) and revised by Zou et al. (2007) was adopted as the criterion tool for validating the new scale. Fundamentally, these represent two distinct yet conceptually related constructs. Children’s Perceived Teacher Trust specifically refers to children’s awareness of their teachers’ confidence in their cognitive abilities, academic competence, moral conduct, and the supportive behaviors derived from such trust (G. Fu, 2025). This perceived trust serves as a critical antecedent variable or constituent element that contributes to positive teacher-student relationships (Sutherland et al., 2024). In contrast, the teacher-student relationship constitutes a fundamental interpersonal dynamic formed through mutual interactions, representing both an underlying relational framework and a comprehensive relational state that influences children’s behavioral patterns, belief systems, and emotional experiences (Aldhafri & Alhadabi, 2019). The Student-Teacher Relationship Scale comprises four dimensions—Closeness, Support, Satisfaction, and Conflict—which collectively characterize an established relational condition. This instrument provides a holistic assessment of the relationship (e.g., “My relationship with the teacher is close and warm”) encompassing both positive aspects (closeness, support, satisfaction) and negative dimensions (conflict). Conversely, the dimensions of Children’s Perceived Teacher Trust are organized around the central theme of “trust,” emphasizing the teacher’s faith in students and subsequent supportive behaviors. This construct represents an interactive process that facilitates relationship development rather than a stabilized relational outcome.

However, a concerning trend suggests a general decline in students’ trust in teachers, often attributed to negative influences from parents, the internet, or teachers themselves (C. Li et al., 2020). To delve deeply into and potentially reverse this trend, the primary prerequisite is possessing a valid instrument capable of accurately measuring student perceptions. Yet, research in this field currently faces significant challenges: Firstly, there is a lack of a clear operational definition for “children’s perception of teacher trust” within the academic community; secondly, its measurement predominantly relies on single-item questions (e.g., “Does your teacher believe in you?”) of questionable reliability and validity (G. Fu, 2025), which are unable to dissect its multidimensional structure; furthermore, the few existing scales are mostly adapted from generalized trust scales designed for adults based on Rotter’s (1967) work, which assume trust is a stable personality trait rather than stemming from the perception of specific behaviors within contextual interactions. This approach struggles to accurately capture primary school students’ trust experiences related to teachers as “significant others.”

Therefore, developing a reliable, valid, and child-centered localized scale is crucial. As Eisenhardt (1989) noted, “when the phenomenon is unclear and existing theories cannot provide a reasonable explanation, employing grounded theory is a matter of course.” This study posits that trust is not an abstract bestowal but is embodied in specific behaviors interpreted by students within daily interactions. Consequently, we adopt the grounded theory qualitative method, aiming to start from the authentic narratives of Chinese primary school students rather than imposing theoretical frameworks based on adult perspectives beforehand, to explore and construct the core dimensions of “perceived teacher trust.” Following this, quantitative methods will be used to rigorously validate the scale’s psychometric properties. This research aims to provide a scientific and effective measurement tool for subsequent studies on the formation mechanisms of teacher-student trust and its impact on student development.

To present the research process more clearly, the study was conducted in two phases: firstly, constructing the scale dimensions through qualitative research using grounded theory, followed by verifying the scale’s reliability and validity through quantitative research involving questionnaire development and refinement.

## 2. Scale Development: A Grounded Theory Approach

### 2.1. Methods

This study employed qualitative interviews and the three-level coding technique of grounded theory to explore middle and upper elementary students (aged 9–12 years) perceptions of teacher trust behaviors and develop a preliminary scale. Based on the principles of purposive and convenience sampling, this study randomly selected 26 students from grades 3 to 5 from the rosters of three public elementary schools in Duyun City, Guizhou Province, China. The sample consisted of 10 third-graders, 8 fourth graders, and 8 fifth graders. In selecting the student sample, we endeavored to maintain balance across grade levels and gender. Additionally, 16 teachers were randomly selected from two of these schools to participate in cluster-based focus group interviews. The sampling procedure adhered to ethical guidelines, obtaining formal approval from the relevant institutional review board, school consent, and informed consent from all participants. 

Grounded theory is particularly well-suited to this purpose, as it addresses common limitations in qualitative research, such as a lack of rigorous methodological support, difficulties in verifying the research process, and limited persuasiveness of conclusions (Yan et al., 2024). Furthermore, collecting multiple data sources throughout the research process facilitates triangulation, enhancing the validity and depth of the findings (Corbin & Strauss, 2015).

Accordingly, this study primarily relied on in-depth interviews with primary school students. To supplement and triangulate these findings, focus group interviews with teachers and relevant online materials were also gathered and analyzed.

### 2.2. Interview Protocols

#### 2.2.1. Primary School Student Interview Protocol

The study involved interviews with a total of 26 elementary school students. Among them, 22 students participated in offline one-on-one interviews: 8 third-grade students (4 girls; M_age_ = 9.45), 7 fourth-grade students (4 girls; M_age_ = 10.32), and 7 fifth-grade students (3 girls; M_age_ = 11.44). Additionally, 4 students (2 girls; M_age_ = 10.41) were interviewed online via WeChat video calls. The average interview duration was approximately 25 min. Data collection for this study primarily involved in-depth interviews with primary school students, supplemented by teacher focus group interviews and online data. A flexible coding strategy was adopted, prioritizing line-by-line coding while incorporating word-by-word and incident-by-incident coding as appropriate. Throughout the coding process, the core principles of classic grounded theory were strictly adhered to, avoiding preconceived notions, categories, or hypotheses to allow them to emerge naturally from the data.

To ensure that the interviewees could understand the interview content more clearly and accurately, the research team—consisting of one professor, two doctoral students, and two primary school head teachers—developed an initial interview protocol through group discussions prior to the formal interviews. Pilot interviews were then conducted with five participants. Based on the outcomes of the pilot interviews and the feedback provided by the participants, the preliminary protocol was revised accordingly, resulting in the final version of the formal interview protocol. The interview protocol included the following questions: (1) Do you feel your teachers trust you? How do they show it? (2) Under what circumstances do you feel most trusted by your teacher? (3) How would you feel if a teacher said to you, “I believe in you”? (4) What specific behaviors from your teacher make you and your classmates feel trusted? Please list a few and rank them according to the level of trust you perceive. (5) Have you ever been dishonest or made a mistake? How did your teacher handle it? (6) What does trust mean to you? (7) What kind of teacher do you trust? Tell me about your favorite teacher.

#### 2.2.2. Teacher Focus Group Interview Protocol

Data obtained from teacher focus group interviews served as crucial Supporting material and validation for this study. To gather rich data, we conducted focus group interviews with a total of 16 teachers (11 females; M_age_ = 35.45) from one urban and one township primary school. The interviews were conducted in two sessions, each lasting approximately two and a half hours.

To ensure that the interviewees could understand the content of the focus group interviews more clearly and accurately, the research team—composed of one professor, two doctoral students, and two primary school head teachers—developed an initial focus group interview protocol through discussion prior to the formal study. Pilot interviews were then conducted with three primary school teachers. Based on the feedback from these pilot interviews and the participants’ suggestions, the initial protocol was revised, resulting in the final version of the formal interview protocol. The interview protocol for teachers included: (1) Please briefly describe your understanding of trust and the relationship between honesty and trust. (2) How do you typically express trust towards your students in the classroom or school environment? What specific behaviors do you engage in? (3) In what situations do students usually demonstrate honest or dishonest behavior, and how do you address it? (4) Have you observed that when you express trust, students are more inclined to be honest? Please provide an example. (5) Describe what you believe are the most effective ways to make students feel trusted.

### 2.3. Data Collection

Interview sessions were audio-recorded and transcribed verbatim shortly after completion. Data collection was suspended when the research team determined that no new conceptual information was emerging from the interviews. The detailed transcripts were coded, and based on these results, a decision was made on whether to resume interviews. This iterative process continued until theoretical saturation was achieved.

Ultimately, twenty-two primary school students were interviewed one-on-one offline, each lasting approximately 25 min on average. The sample included 8 third graders (4 girls; M_age_ = 9.45), 7 fourth graders (4 girls; M_age_ = 10.32), and 7 fifth graders (3 girls; M_age_ = 11.44). Additionally, focus group interviews were conducted with 16 teachers (11 females; M_age_ = 35.45) from two primary schools. Furthermore, 2 teachers (1 females; M_age_ = 32.22) and 4 students (2 girls; M_age_ = 10.41) were interviewed online via WeChat video calls to supplement the data. The extensive availability of online materials concerning teacher-student trust provided a crucial source for triangulation. This approach aligns with prior research utilizing online content for construct development (Dulfer et al., 2024). Searches were conducted on popular Chinese platforms (e.g., CNKI, Xiaohongshu, Weibo, Sohu) using keywords such as “primary school teacher-student trust behavior.” The initial search yielded approximately 1.05 million results. After removing irrelevant and duplicate content, 12 relevant text documents were selected for analysis, comprising approximately 21,000 Chinese characters.

### 2.4. Coding Process

#### 2.4.1. Open Coding

The data analysis employed the three-level coding procedures of grounded theory. Specifically, during the first-level coding stage—open coding—segments relevant to children’s perception of teacher trust were extracted and coded from the raw materials. Initially, two researchers collaboratively reviewed all textual data to identify and label semantic segments pertaining to the research topic. A coding system was adopted to indicate the source of raw data: “I” for individual interviews, “G” for questionnaires, and “N” for online materials. The first digit following the letter indicated the sequential identifier of the data source, and the second digit represented the order of the semantic segment within that source. For example, “I05-2” refers to the second semantic segment from the fifth interviewee. Since two rounds of teacher focus group interviews were conducted in this study, “GA” was used to denote the first session and “GB” the second.

To facilitate accurate interpretation of each semantic segment with reference to the original context and to reduce coding subjectivity, all segments were organized according to the source codes (I, G, N). Two researchers performed the initial coding independently. Throughout this process, the researchers maintained an open attitude and prioritized in vivo codes—unique expressions used by the participants themselves or verbatim descriptions from online materials. After completing independent coding, the two researchers compared, discussed, screened, and integrated the codes through a consensus-building process to finalize the initial codes (see Table 1). Given the fragmented nature of these initial codes, they were further screened, categorized, and integrated. Codes with similar meanings were merged; for instance, “stopping to explain slowly for us” and “willing to stop and explain problems we didn’t understand” were combined into a single code labeled “Academic Guidance.” Ultimately, this process yielded 489 semantic segments, which were condensed into 286 initial codes and further abstracted into 78 initial concepts.

#### 2.4.2. Selective Coding

The secondary coding phase, known as axial or selective coding, commenced after the emergence of core categories. Guided by principles of centrality, explanatory power, recurrence, and the ability to form meaningful connections with other variables (Jia & Tan, 2010), the 78 initial concepts were systematically analyzed and compared. Through this process, conceptually similar codes were integrated, resulting in the consolidation of the initial concepts into 18 subcategories. After several rounds of discussion, these 18 subcategories were further synthesized into 4 core categories (see Table 2).

#### 2.4.3. Theoretical Coding

Theoretical coding refers to the interrelationships—such as causal, parallel, or sequential connections—among concepts or categories formed through substantive coding (Jia & Tan, 2010). To apply theoretical coding to primary school students’ perception of teacher trust, it is first necessary to clarify what constitutes the perception of teacher trust within the school context. Allport, the founder of personality trait theory (Rosenzweig & Fisher, 1997), proposed that “an individual’s activity tendencies consist partly of what is variable and partly of what remains stable.” The “variable part” corresponds to the “state,” which refers to specific psychological conditions and behaviors potentially elicited by stimuli in the environment. The “stable and invariant” aspect constitutes the “trait”—a generalized and focused neuropsychological structure that exhibits individual differences and serves to activate and guide adaptive and expressive behavior (Yan et al., 2024).

**Figure 1:**
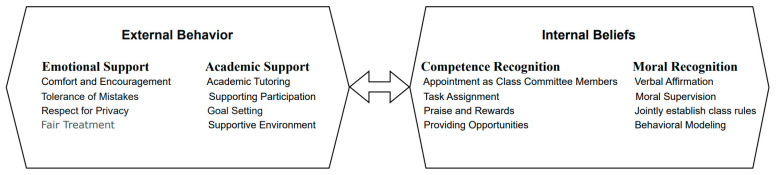
Conceptual model of perceived teacher trust behavior among primary school students.

Integrating our coding results and comparative analysis of the emergent concepts, we propose that the perception of teacher trust is influenced both by variable, “state”-like external behaviors and stable, “trait”-like internal beliefs across different contexts. Therefore, perceived teacher trust can be defined as a student’s perception of the teacher’s support in emotional and academic domains, as well as recognition of the student’s capabilities and conduct (see Figure 1).

External behaviors encompassed Emotional Support and Academic Support. Emotional Support was manifested through comforting and encouragement during difficulties, forgiveness for mistakes, respect for privacy, and fair treatment. Academic Support was evident in behaviors such as learning guidance, support for competitions, help with setting learning goals, and adapting the learning environment. Internal beliefs comprised Competence Recognition and Moral Recognition. Competence Recognition was demonstrated through appointments as class cadres, assignment of tasks, acknowledgment of achievements (grades/scores), and providing opportunities for self-expression. Moral Recognition was primarily reflected in verbal affirmation of moral conduct, entrusting students with moral supervision roles, involving them in establishing class rules, and using them as behavioral exemplars.

### 2.5. Theoretical Saturation Check

Upon completing the initial coding process, the research team conducted supplementary interviews with two additional students and two teachers. A comprehensive analysis of this new data, following the same coding procedures, yielded no new categories or concepts. The absence of novel information in this subsequent data collection indicated that the theoretical structure of the Perceived Teacher Trust Behavior Scale for primary school students had reached theoretical saturation.

## 3. Scale Development and Validation

### 3.1. Subjects and Methods

#### 3.1.1. Subjects

Using on-site cluster sampling, a collective questionnaire survey was administered to 1400 students in grades 3–5 from three schools in Guizhou Province. The collected data were randomly split into two halves: one for exploratory factor analysis and the other for confirmatory factor analysis (Anderson & Gerbing, 1988; Robert & Browne, 1983). Questionnaires with incomplete or patterned responses were excluded as invalid. A total of 1317 valid questionnaires were retained, resulting in a valid response rate of 94%. The sample included 396 third graders, 469 fourth graders, and 452 fifth graders; 700 boys and 617 girls; with a mean age of 10.25 years.

The total of 1317 valid samples were randomly divided into two approximately equal subsamples. Subsample 1 (*n* = 656) was used for item analysis and exploratory factor analysis of the Perceived Teacher Trust Behavior Scale, while Subsample 2 (*n* = 661) was utilized for confirmatory factor analysis and reliability/validity testing. The test of measurement invariance required the use of the entire sample of 1317 participants to ensure sufficient statistical power across subgroups.

#### 3.1.2. Questionnaire Development and Revision

Based on the conceptual framework of primary school students’ perception of teacher trust derived from grounded theory and interview coding data, 32 descriptive statements were initially generated, with each subcategory corresponding to two items. To ensure the content validity of the preliminary scale, an expert panel was convened, consisting of one psychology professor, two doctoral students, and three primary school principals. The panel evaluated the relevance, clarity, and appropriateness of each item in relation to the theoretical dimensions derived from the grounded theory analysis. The evaluation was conducted through structured discussions during a meeting, with the aim of reaching full consensus. Each expert provided a binary judgment (‘agree’ or ‘disagree’) on whether the items accurately reflected their in-tended dimensions and whether the wording was suitable for the target population. All items were reviewed through iterative discussions until full consensus was achieved. This consensus-based approach aligns with the principles of grounded theory, ensuring that the items remained grounded in the raw data rather than the re-searchers’ preconceptions. Following this process, items were deleted, merged, or modified, resulting in a final scale of 16 items (e.g., ‘When I make a mistake, the teacher guides me patiently’; ‘The teacher assigns me important tasks’), with four items corresponding to each dimension. 

To enhance the precision of the scale’s wording, 30 primary school students were invited to complete the questionnaire prior to formal administration. Based on their feedback, the item descriptions were further refined, resulting in the pilot version. The pilot questionnaire consisted of two sections: the first collected demographic information (e.g., age, grade, gender), and the second included a 16-item 5-point Likert scale. Responses were scored from 1 (“never”) to 5 (“always”). All items were positively scored, and the total scale score was calculated by summing all item scores, with higher scores indicating a higher level of perceived teacher trust by children.

#### 3.1.3. Criterion Tool

The Student-Teacher Relationship Scale (STRS), developed by Pianta (2001) and revised by Zou et al. (2007), was adopted as the criterion measure. This scale consists of 23 items rated on a 5-point Likert scale, ranging from “definitely not” (1 point) to “definitely yes” (5 points). Higher total scores indicate better quality of student-teacher relationships. The scale comprises four dimensions: closeness, support, satisfaction, and conflict. It demonstrates good reliability and validity, with a Cronbach’s α coefficient of 0.89 in the present study.

#### 3.1.4. Statistical Data Processing

In the present study, we performed item analysis and exploratory factor analysis (EFA) using SPSS 30.0, while confirmatory factor analysis (CFA) and measurement invariance testing were conducted with Mplus. Additionally, the Conflict dimension in the Teacher-Student Relationship Scale was reverse-coded.

## 4. Results

### 4.1. Item Analysis

Based on the total scores of the Perceived Teacher Trust Scale, Sample 1 was divided into high- and low-score groups using the 27th and 73rd percentiles of the total score (Lahza et al., 2023). The low-score group comprised 184 participants (28.0%), and the high-score group consisted of 182 participants (27.7%). Independent samples *t*-tests were conducted on all 16 items of the PT scale to examine their discriminatory power. The results indicated that all 16 items showed statistically significant differences between the high- and low-score groups (*p* < 0.001), demonstrating satisfactory discriminatory power for each item.

Internal consistency reliability analysis was conducted on the 16 items of the Perceived Teacher Trust Scale. The results indicated an overall Cronbach’s α coefficient of 0.885. However, three items (PT3, PT6, and PT12) demonstrated relatively low item-total correlations (correlation coefficients < 0.40). Furthermore, the deletion of these items resulted in an increase in the α coefficient. Consequently, the removal of these three items may be considered to enhance the overall reliability of the scale (Carpenter, 2018), with final decisions regarding their retention to be further informed by subsequent exploratory factor analysis results.

### 4.2. Exploratory Factor Analysis

First, Bartlett’s test of sphericity (*χ*^2^ = 3790.120, *df* = 120, *p* < 0.001) and the Kaiser–Meyer–Olkin (KMO) measure (KMO = 0.916) were conducted on Sample 1. The results indicated that the items were not mutually independent and likely shared common underlying factors, supporting the suitability of factor analysis. Using principal axis factoring, four factors were extracted (the number of factors is determined based on parallel analysis, the number of factors in qualitative research, and the principle that the eigenvalue is greater than 1), accounting for a cumulative variance of 59.749%. Specifically, the first factor explained 37.468% of the variance, the second factor explained 8.222%, the third explained 7.116%, and the fourth explained 6.943%. An analysis of the communalities of each item revealed that PT3 (0.075), PT6 (0.184), and PT12 (0.155) all had communalities below 0.2, indicating that these items were not adequately explained by the extracted factors (Boateng et al., 2018). Therefore, it was decided to eliminate these three items.

After the removal of PT3, PT6, and PT12, an exploratory factor analysis (EFA) was conducted on the remaining 13 items. The results of Bartlett’s test of sphericity (*χ*^2^ = 3521.50, *df* = 78, *p* < 0.001) and the Kaiser–Meyer–Olkin (KMO) measure (KMO = 0.910) indicated that the data were highly suitable for factor analysis. Four factors were extracted using principal axis factoring with promax rotation. The results of the factor analysis are presented in Table 3, showing a cumulative variance explained of 69.451%. The variance explained by each factor was as follows: Factor 1 accounted for 43.539%, Factor 2 for 9.835%, Factor 3 for 8.496%, and Factor 4 for 7.581%. In this second analysis, the factor loading pattern was more distinct and interpretable: Factor 1 was primarily defined by PT13, PT14, PT15, and PT16, with loadings ranging from 0.605 to 0.775, and was labeled Competence Recognition. Factor 2 was primarily defined by PT1, PT2, and PT4, with loadings ranging from 0.688 to 0.820, and was labeled Emotional Support. Factor 3 was primarily defined by PT9, PT10, and PT11, with loadings ranging from 0.647 to 0.836, and was labeled Academic Support. Factor 4 was primarily defined by PT5, PT7, and PT8, with loadings ranging from 0.499 to 0.790, and was labeled Moral Recognition.

In the secondary analysis, the communalities of all retained items ranged from 0.442 to 0.655, indicating that these items were adequately explained by the extracted factors. Through two rounds of exploratory factor analysis, a final structure of the PT scale comprising 13 items and 4 factors was established, thereby laying the groundwork for subsequent confirmatory factor analysis.

### 4.3. Confirmatory Factor Analysis 

**Figure 2:**
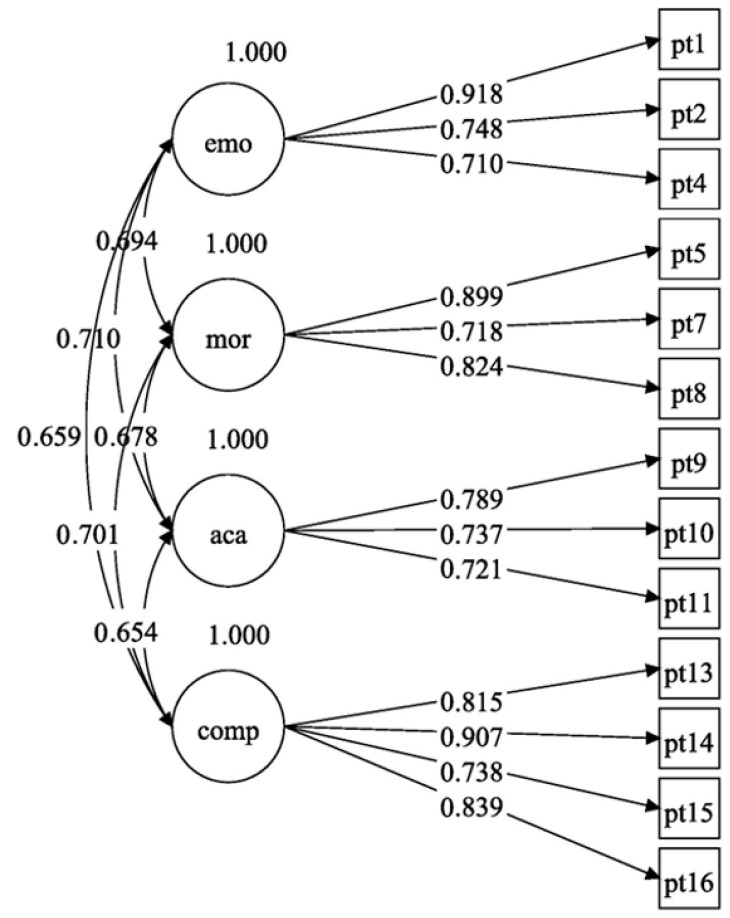
Results of factor loadings and correlations among latent variables in the CFA. Note. comp = Competence Recognition, emo = Emotional Support, aca = Academic Support, mor = Moral Recognition.

Based on the results of the previous exploratory factor analysis, this study constructed a confirmatory factor analysis model using the final 13 items (see Appendix A) to examine the validity of the proposed four-factor structure. The confirmatory factor analysis conducted on Sample 2 indicated that the model exhibited a good statistical fit. An analysis of the factor loadings of these 13 items revealed strong associations between each latent variable and its corresponding items, as illustrated in Figure 2. Specifically, the factor loadings ranged from 0.710 to 0.918 for the emotional support dimension, from 0.718 to 0.899 for the moral recognition dimension, from 0.718 to 0.899 for the academic support dimension, and from 0.738 to 0.907 for the competence recognition dimension. These results demonstrate that the optimized measurement model, refined through two rounds of exploratory factor analysis, effectively accounts for the variance in the data, with the indicators of each dimension appropriately reflecting the characteristics of their respective latent variables. The correlations among the four latent variables ranged between 0.654 and 0.710.

All model fit indices met the statistically acceptable standards (Goretzko et al., 2024). As shown in Table 4, the CFA model with a 4-factor structure had *χ*^2^*/df* = 3.376, *RMSEA* = 0.060, *SRMR* = 0.027, *TLI* = 0.980, and *CFI* = 0.985. These fit indices demonstrate that the 4-factor model fits the sample data very well. Furthermore, based on the magnitude of correlations, we sequentially merged Emotional Support, Academic Support, Moral Recognition, and Competence Recognition to construct CFA models with different numbers of factors (Model 1, 2, 3, 4). The results indicated that Model 1 (the 4-factor model) had the best fit. Model 2 (the 3-factor model) had an *RMSEA* value of 0.085, which exceeded the critical value of 0.08. The fit indices of Model 3 and Model 4 deteriorated further. Therefore, it can be concluded that the four-factor structure was the model that best represented the factor structure of the sample data among all the candidate models.

### 4.4. Convergent and Discriminant Validity

Regarding convergent validity, the Average Variance Extracted (AVE) values were as follows: competence recognition (0.684), moral recognition (0.668), academic support (0.532) and emotional support (0.635)—all above the critical value of 0.50, indicating good convergent validity for these dimensions (DeVellis & Thorpe, 2021).

The results of the discriminant validity analysis are presented in Table 5. The $\sqrt{\mathrm{AVE}}$ values for the four dimensions—emotional support, moral recognition, academic support, and competence recognition—were 0.757, 0.781, 0.709, and 0.793, respectively. According to the Fornell-Larcker criterion (Collier, 2020), each $\sqrt{\mathrm{AVE}}$ value should be greater than the correlation coefficients between dimensions. The results indicate that all correlation coefficients are lower than the corresponding √(“AVE”), demonstrating adequate discriminant validity among the latent variables according to the Fornell-Larcker criterion. Nonetheless, the Heterotrait-Monotrait Ratio (HTMT) analysis showed that all HTMT values were below the stringent threshold of 0.85 (Henseler et al., 2015). Based on the fit indices of CFA models with different factor structures presented in Table 4, as well as the results of the Fornell-Larcker criterion and the HTMT values, it can be concluded that the four subscales demonstrate acceptable discriminant validity.

### 4.5. Criterion-Related Validity Test

The Teacher-Student Relationship Scale revised by Zou et al. (2007) was employed as the criterion tool to examine the criterion-related validity using data from Sample 2. The results, as presented in Table 6, indicate that the Children’s Perceived Teacher Trust Scale demonstrated statistically significant correlations with both the total score and all subdimensions of the Teacher-Student Relationship Scale at the 0.01 level. Specifically, the correlation coefficient between the total score of the Children’s Perceived Teacher Trust Scale and the total score of the Teacher-Student Relationship Scale was 0.489, indicating a moderate correlation. Furthermore, correlations with individual subdimensions generally exceeded 0.30, among these, the Conflict subscale of the Teacher-Student Relationship Scale was reverse-coded, indicating satisfactory criterion-related validity.

### 4.6. Reliability Testing

Following item analysis, exploratory factor analysis, and confirmatory factor analysis, the Perceived Teacher Trust Scale retained 13 items. In terms of reliability (Table 7), the Composite Reliability, Cronbach’s alpha and McDonald’s ω coefficients for all four subdimensions reached acceptable levels (>0.70). The competence recognition subdimension demonstrated the highest reliability (CR = 0.896, α = 0.870, ω = 0.874), followed by moral recognition (CR = 0.857, α = 0.824, ω = 0.827), while the academic support subdimension, though relatively lower, remained within the acceptable range (CR = 0.793, α = 0.751, ω = 0.753).

Furthermore, split-half reliability analysis conducted on Sample 2 resulted in an unequal-length Spearman–Brown coefficient of 0.847, exceeding the critical value of 0.7 (Harrison et al., 2020), indicating satisfactory split-half reliability.

### 4.7. Measurement Invariance

The complete set of 1317 valid samples was utilized for invariance testing. The sample consisted of 700 males and 617 females, with 396 third graders, 469 fourth graders, and 452 fifth graders. Multi-group confirmatory factor analysis was conducted to examine the measurement invariance of the Children’s Perceived Teacher Trust Scale across gender and grade levels. Following established criteria (Byrne, 2016), the results presented in Table 8 and Table 9 demonstrate that all three models—configural invariance (the same factor structure holds across groups), metric invariance (factor loadings are equal across groups, establishing a common measurement unit for the latent factors) and scalar invariance (item intercepts are equal across groups, enabling meaningful comparisons of latent factor means) exhibited good fit. The criteria for measurement invariance were satisfied, as the changes in fit indices (Δ*RMSEA*, Δ*CFI*, and Δ*SRMR*) were all below 0.050. These findings indicate that the Children’s Perceived Teacher Trust Scale demonstrates full measurement invariance across both gender and grade levels.

## 5. Discussion

Our research project documented the trust experiences of upper elementary school students toward their teachers, culminating in the development of a novel Perceived Teacher Trust Behavior Scale. Through the successful validation of this multidimensional interpersonal trust measure, our study contributes to the existing literature in several significant aspects: This scale represents the first and only instrument developed through grounded theory and qualitative interviews with children to establish theoretical dimensions, specifically designed to measure teacher-triggered trust behaviors among elementary students. This distinguishes it from previous interpersonal trust measures, such as Interpersonal Trust Scale (Rotter, 1967), Children’s General Trust Beliefs scale (Rotenberg et al., 2005). Meanwhile, most existing interpersonal trust scales have been adapted from Rotter’s (1967) general trust scales designed for adults, which operate under the premise that trust constitutes a stable personality trait rather than originating from the perception of specific behaviors within contextual interactions. The scale most closely aligned with our research is the Children’s Generalized Trust Beliefs Scale developed by Rotenberg et al. (2005), designed to assess children’s trust levels toward four categories of individuals (father, mother, teacher, and peer). However, it does not specifically measure children’s perception of adults’ trust in themselves. The item pool targeting teacher trust contains only six items, such as “The teacher told Suzy’s class that they were going to see a video instead of having a math lesson. The teacher said the video was lost. How likely is it that the video was lost?” Some measurements rely solely on single-item questions (e.g., “Does your teacher have confidence in your learning ability? ① Yes, ② No”) (G. Fu, 2025).

Furthermore, we have established perceived teacher trust behavior as a multidimensional construct comprising four latent factors, with this structure demonstrating measurement invariance across different gender and grade subgroups.

We thoroughly examined the reliability and validity of the Perceived Teacher Trust Behavior Questionnaire. Reliability assessment primarily utilized internal consistency reliability and split-half reliability as indicators. Results indicated that the questionnaire’s Cronbach’s α coefficients ranged between 0.75 and 0.87, and the Spearman–Brown coefficient for split-half reliability was 0.847, demonstrating good reliability. Validity was evaluated using multiple indicators, including construct validity, criterion-related validity, convergent validity, and discriminant validity. The results of confirmatory factor analysis (CFA) showed that the four-factor model achieved the best fit, with all items loading strongly on their intended dimensions. Significant positive correlations between teacher–student relationships and perceived teacher trust—including each of its dimensions—provided evidence of good criterion-related validity. In terms of convergent validity, the average variance extracted (AVE) for all four dimensions exceeded the critical value of 0.50, indicating adequate convergent validity. Discriminant validity was also supported, confirming that the four dimensions are distinct from one another. These collective findings demonstrate that the questionnaire possesses robust psychometric properties.

Perceived teacher trust exerts a significant influence on students’ psychological development (Xu et al., 2025), academic enhancement (Aldhafri & Alhadabi, 2019), and teacher-student relationships (Sutherland et al., 2024). Through the application of grounded theory, qualitative interviews, and psychometric validation of scales, we operationalize perceived teacher trust as students’ perceived trust from their teachers. Perceived teacher trust refers to students’ perceptions of trust from their teachers, primarily encompassing four dimensions: emotional and academic support, as well as recognition of competence and moral conduct. These four dimensions of the scale broadly cover the “external behaviors” and “internal beliefs” of primary school students’ perceived teacher trust identified through interviews. External behaviors include emotional and academic support, while internal beliefs comprise recognition of competence and moral conduct.

Although this study systematically explored the concept and structural dimensions of primary school students’ perceived teacher trust, certain limitations should be acknowledged. First, the scale is designed for middle and upper elementary students (aged 9–12 years) and may not be appropriate for lower elementary students (under 9 years old). Younger students may have difficulty fully comprehending all items in the item pool due to their limited cognitive abilities and school life experience. Secondly, the use of qualitative interviews and the grounded theory approach inevitably introduced some subjectivity. Although multiple independent coders and repeated discussions were employed to minimize subjective bias, future research could enhance objectivity by expanding sample sizes and incorporating more diverse literature. Moreover, some items exhibited cross-loading issues across theoretical dimensions. Future studies could refine item design to improve discriminant validity between dimensions. Additionally, the sample data were primarily sourced from specific regions, limiting representativeness. The generalizability of the findings needs to be validated across broader geographical contexts. Future research should expand the sample to enhance widespread applicability and precision. Finally, although the study targeted upper primary school students, their abstract logical thinking is still developing. Therefore, items must align with their cognitive level, using simple and understandable language. Overly fine-grained dimensions may lead to confusion during response collection.

## 6. Conclusions

The Primary School Students’ Perceived Teacher Trust Scale, developed based on the school context and children’s perspectives, identified four dimensions through qualitative interviews and grounded theory. The PTTBS consists of four factors. Factor 1, Emotional Support, is primarily manifested in teachers’ comforting and encouraging students when they face difficulties, showing tolerance and understanding when students make mistakes, and respecting and protecting students’ privacy. Factor 2, Recognition of Moral Conduct, is reflected by teachers verbally affirming students’ moral behavior, entrusting them with moral supervision roles in the class, involving them in co-establishing class rules, and recognizing exemplary behavior through modeling. Factor 3, Academic Support, is demonstrated through teachers assisting students with homework guidance, subject competitions, setting learning goals, and creating a supportive classroom environment. Factor 4, Recognition of Competence, is evidenced by teachers appointing students as class leaders, assigning academic and classroom tasks, rewarding achievements in competitions and exams, and providing various opportunities for students to showcase their abilities.

The scale underwent rigorous validation, including exploratory and confirmatory factor analyses, as well as reliability and validity tests. After excluding substandard items (Items 3, 6, and 12), the final scale retained 13 items. The results indicate that the scale exhibits satisfactory internal consistency reliability, construct validity, and criterion-related validity, meeting psychometric standards. This study provides a practical tool for theoretical research and practical applications related to primary school students’ perceived teacher trust.

## Figures and Tables

**Table 1:** Initial coding example.

Source Material (Third-Grade Child Interview)	Open Coding
	Initial Coding (Initial Concept)
I have a very good relationship with my teacher, and the teacher trusts me a lot. I think the fact that the teacher asked me to serve as a class cadre shows trust in me [I19-1].	[I19-1] The teacher asked me to be a class cadre
(Are you currently a class cadre?) Yes, I am a group leader and the subject representative for Chinese and English. (Does collecting homework make you feel particularly trusted by the teacher?) Yes [I19-2].	[I19-2] Collecting homework makes me feel trusted
(What behaviors of the teacher made you feel trusted? For example.) Once, a classmate kicked a soccer ball that hit me, and the teacher came over and comforted me [I19-3].	[I19-3] The teacher came over and comforted me
Another time, during the monthly exam, I didn’t achieve the score I wanted, and the teacher said if I wanted to aim for a higher score, I could go to her office for tutoring [I19-4].	[I19-4] Didn’t achieve the score I wanted
There was also a time when the teacher asked me to lead my classmates in reading English [I19-5].	[I19-5] Asked me to teach classmates to read English
I like the English teacher because whenever we don’t understand an English word, she always pauses and explains it to us slowly [I19-6]. (What kind of teacher do you like?) I like teachers who are gentle and willing to pause to explain the problems we don’t understand [I19-6].	[I19-6] Pauses and explains to us slowly [I19-6] Willing to pause and explain problems we don’t understand
And the math teacher, when explaining math problems—if many students couldn’t solve it, and one person got it right and was expecting praise—the teacher wouldn’t praise them immediately. Instead, she would finish explaining the problem to the whole class first and then announce to everyone who had gotten it right [I19-7].	[I19-7] The teacher finishes explaining the problem first and then praises
(Is the teacher asking you to help collect homework, supervise class assignments, or erase the blackboard a sign of trust?) Yes [I19-8].	[I19-8] Helping the teacher collect homework is a sign of trust
(Which of these indicates a higher level of trust?) Having us supervise the class [I19-9].	[I19-9] Supervise the class
(Is helping the teacher carry their phone a sign of trust?) Yes, but it’s better not to help with that. It’s preferable to just carry homework, because the phone might already have some damage, or if the teacher’s personal item has a slightly broken part, and you carry it, the teacher might forget and blame you for the damage [I19-10].	[I19-10] Help the teacher carry their phone
(What else would you be willing to help the teacher with?) Maintaining discipline. I feel very happy when the teacher trusts me [I19-11] …	[I19-11] I feel very happy when the teacher trusts me…

**Table 2:** Selective coding (Excerpt).

Source Material Semantic Segments	Open Coding		
	Initial Coding (Initial Concept)	Subcategory	Core Category
The teacher trusts me a lot. Sometimes during class when a classmate pulls my hair and I don’t make a sound, the teacher will come over, gently rub my head to comfort me, hug me, and then criticize him [I01-1].	Trust and comfort	Comfort and Encouragement	
When I was little, I did poorly on a test paper. I didn’t get it signed and lied to the teacher about it. Later, I told the teacher the truth. The teacher patiently said it’s okay to make mistakes, just be more careful next time [I16-8].	The teacher didn’t scold me.	Tolerance of Mistakes	Emotional Support
Some people in our class always shift the blame onto me when they do something wrong. I don’t know why, but I feel it’s unfair [I07-7].	It’s not fair to put the blame	Fair Treatment	
When I communicate with her, first I must affirm the parts she got right herself. I need to know how to respect her privacy, keep things confidential, and protect the child’s self-esteem [GB06-2].	Respect for Privacy	Respect for Privacy	
Some children, even if their grades aren’t good, have a good attitude and are very honest. With these children, we should encourage them more and affirm their positive moral qualities. There’s one child in our class who is especially well-behaved, and I often praise him in class for being an honest child [N03-9].	Moral Recognition	Verbal Affirmation	
Sometimes the teacher asks me to be an integrity monitor to see if classmates are lying [I02-4].	Integrity supervision	Moral Supervision	Moral Recognition
I feel the teacher asking me to do things shows trust in me. This time, I discussed class rules with the teacher along with other class cadre students. I told the teacher that swearing is not allowed [I03-7].	Establish class rules	Jointly establish class rules	
The most frequent way is using me as an example for the classmates, saying I have a serious attitude towards work and good grades, so (the teacher) asks me to help him/her do things [I11-5].	Take me as an example	Behavioral Modeling	
At school, whenever I get a relatively low score, (the teacher) would ask me to go to the office and then give me tutoring there [I04-3].	give me some advice to help me improve my grades	Academic Tutoring	
For example, previously, our Teacher Wu said, “Your math must be very good.” I like math, so she had me participate in a math competition. She trusted my math ability [I18-3].	Let me participate in the math competition	Supporting Participation	Academic Support
For example, previously, our Teacher Wu said, “Your math must be very good.” I like math, so she had me participate in a math competition. She trusted my math ability [I18-3].	let’s do even better	Goal Setting	
(She) moved me from the back row to the front row, and also, when I couldn’t see (the blackboard), she would let me come up to write (on it). Actually, I’ve forgotten a lot of it; it seems the teacher has helped me a great deal [I15-2].	position adjustment	Supportive Environment	
I like the Chinese teacher more. She keeps her promises, like last time she said she would give praise and prizes if we did well on the test, and she always does [I14-8].	Praise and Rewards	Praise and Rewards	
The teacher trusts me a lot. I think the fact that the teacher asked me to be a class cadre shows trust in me. I am a group leader and the Chinese and English subject representative [I19-1].	Appointment as Class Committee Members	Appointment as Class Committee Members	Competence Recognition
Helping the teacher with tasks is a form of trust, like collecting homework. I feel that collecting homework shows more trust in us because the teacher trusts that we won’t secretly remove someone’s homework and pretend it was turned in. The teacher cares most about homework collection [I18-13].	Help the teacher with something	Task Assignment	
Start from the student’s interests, and simultaneously provide them with opportunities to showcase themselves. There was a classmate in our class who didn’t speak. I noticed he was quite good at drawing. So for this Children’s Day, I gave him a picture to copy, and he did very well. I praised him. Later, in my classes, he started taking notes seriously and listening attentively [GA08-3]... (Total of 489 entries)	Providing Opportunities	Providing Opportunities	
	… (Total of 78 entries)	… (Total of 18 entries)	… (Total of 4 entries)

**Table 3:** Results of exploratory factor analysis after item deletion.

Items	Competence Recognition	Emotional Support	Academic Support	Moral Recognition	Communality
PT1: Even when my performance is unsatisfactory, the teacher continues to believe that I am capable of doing well next time.	−0.100	**0.688**	0.171	0.086	0.655
PT2: When I make a mistake, the teacher provides patient guidance.	0.084	**0.783**	−0.093	−0.047	0.559
PT4: The teacher will keep my secret safe from others.	−0.007	**0.820**	−0.046	−0.089	0.534
PT5: The teacher said that I am a child with good conduct.	−0.021	0.214	0.171	**0.499**	0.605
PT7: The teacher involves me in the development of our class code of conduct.	0.009	−0.073	−0.097	**0.763**	0.442
PT8: The teacher encourages classmates to emulate my positive behavioral conduct.	0.022	−0.054	0.004	**0.790**	0.592
PT9: When encountering challenging problems, the teacher provides patient guidance.	−0.030	0.040	**0.728**	−0.031	0.516
PT10: The teacher supports my participation in various academic competitions.	−0.001	−0.054	**0.836**	−0.067	0.581
PT11: The teacher encourages me to establish learning goals.	0.111	−0.051	**0.647**	0.026	0.489
PT13: The teacher appoints me to class leadership roles (e.g., group leader).	**0.750**	−0.072	−0.019	0.034	0.518
PT14: The teacher entrusts me with important responsibilities.	**0.605**	0.219	−0.024	0.078	0.639
PT15: The teacher acknowledges my progress and achievements.	**0.696**	0.010	0.026	−0.045	0.476
PT16: The teacher provides me with various opportunities for self-expression	**0.775**	−0.021	0.056	−0.013	0.619
Eigenvalue	5.660	1.279	1.104	0.986	-
Var. Explained %	43.539	9.835	8.496	7.581	-
Cumulative Var. %	43.539	53.374	61.870	69.451	-

Note: Bolded values in the table indicate the highest factor loading for each item on its respective factor.

**Table 4:** Confirmatory factor analysis fit indices.

Models	*χ* ^2^	*df*	*χ* ^2^ */df*	*RMSEA*	*SRMR*	*TLI*	*CFI*
Model 1: 4 factors (emo, mor, aca, comp)	199.162	59	3.376	0.060	0.027	0.980	0.985
Model 2: 3 factors (emo + aca, mor, comp)	357.427	62	5.765	0.085	0.039	0.959	0.968
Model 3: 2 factors (emo + aca + mor, comp)	616.040	64	9.626	0.114	0.052	0.927	0.940
Model 4: Single factor (emo + aca + mor + comp)	1083.498	65	16.669	0.154	0.069	0.867	0.889
Recommended Thresholds	-	-	2~5	≤0.08	≤0.06	≥0.90	≥0.90

**Table 5:** Results of discriminant validity analysis.

Variables	Emotional Support	Moral Recognition	Academic Support	Competence Recognition
Emotional Support	**0.** **797**	0.674	0.692	0.649
Moral Recognition	0.694	**0.** **817**	0.670	0.688
Academic Support	0.710	0.678	**0.** **729**	0.645
Competence Recognition	0.659	0.701	0.654	**0.** **827**

Note: The bold figures on the diagonal represent the $\sqrt{\mathrm{AVE}}$ values; the lower triangle contains the correlation matrix among the four sub-dimensions, and the upper triangle displays the HTMT values between the variables.

**Table 6:** Analysis of criterion-related validity between the Perceived Teacher Trust Behavior scale (PTTBS) and the teacher-student relationship scale (TSRS).

Variable	TSRS	Closeness	Conflict	Satisfaction	Support
PTTBS	0.487 **	0.396 **	0.361 **	0.409 **	0.410 **
Emotional Support	0.407 **	0.325 **	0.301 **	0.353 **	0.318 **
Moral Recognition	0.382 **	0.324 **	0.271 **	0.319 **	0.340 **
Academic Support	0.382 **	0.297 **	0.293 **	0.314 **	0.329 **
Competence Recognition	0.414 **	0.339 **	0.308 **	0.346 **	0.347 **

Note: ** indicates *p* < 0.01.

**Table 7:** Results for Reliability.

Sub-Scales	Composite Reliability	Cronbach’s α	McDonald’s ω
Emotional Support	0.838	0.801	0.805
Moral Recognition	0.857	0.824	0.827
Academic Support	0.793	0.751	0.753
Competence Recognition	0.896	0.870	0.874

**Table 8:** Measurement invariance analysis across gender.

Model	*χ* ^2^	*df*	Δ*df*	Δ*χ*^2^	*RMSEA*	*CFI*	*SRMR*	Δ*RMSEA*	Δ*CFI*	Δ*SRMR*
1	426.578	118	-	-	0.063	0.981	0.029	-	-	-
2	444.698	127	9	29.995	0.062	0.980	0.030	−0.001	−0.001	0.001
3	456.364	162	35	34.387	0.053	0.981	0.030	−0.009	0.001	0.000

Note: Models 1 to 3 represent the configural invariance model, metric invariance (weak invariance) model, and scalar invariance (strong invariance) model, respectively.

**Table 9:** Measurement invariance analysis across grade level.

Model	*χ* ^2^	*df*	Δ*df*	Δ*χ*^2^	*RMSEA*	*CFI*	*SRMR*	Δ*RMSEA*	Δ*CFI*	Δ*SRMR*
1	492.004	177	-	-	0.064	0.980	0.031	-	-	-
2	485.607	195	18	20.976	0.058	0.982	0.032	−0.006	0.002	0.001
3	521.321	265	70	58.449	0.047	0.984	0.033	−0.011	0.002	0.001

## Data Availability

The data are available on request from the corresponding author. The data are not publicly available due to privacy or ethical restrictions.

## Appendix A

**Table A1:** The Final Verified Qualified Scale.

Items	Never	A Few	Sometimes	Usually	Always
PT1: Even when my performance is unsatisfactory, the teacher continues to believe that I am capable of doing well next time.	1	2	3	4	5
PT2: When I make a mistake, the teacher provides patient guidance.	1	2	3	4	5
PT3: The teacher will keep my secret safe from others.	1	2	3	4	5
PT4: The teacher said that I am a child with good conduct.	1	2	3	4	5
PT5: The teacher involves me in the development of our class code of conduct.	1	2	3	4	5
PT6: The teacher encourages classmates to emulate my positive behavioral conduct.	1	2	3	4	5
PT7: When encountering challenging problems, the teacher provides patient guidance.	1	2	3	4	5
PT8: The teacher supports my participation in various academic competitions.	1	2	3	4	5
PT9: The teacher encourages me to establish learning goals.	1	2	3	4	5
PT10: The teacher appoints me to class leadership roles (e.g., group leader).	1	2	3	4	5
PT11: The teacher entrusts me with important responsibilities.	1	2	3	4	5
PT12: The teacher acknowledges my progress and achievements.	1	2	3	4	5
PT13: The teacher provides me with various opportunities for self-expression	1	2	3	4	5

NOTE: PT1, PT2 and PT3 define Factor “Emotional Support”; PT4, PT5 and PT6 define Factor “Moral Recognition”; PT7, PT8 and PT9 define Factor “Academic Support”; PT10, PT11, PT12 and PT13 define Factor “Competence Recognition”.
